# Tryptophan-kynurenine metabolism: a link between the gut and brain for depression in inflammatory bowel disease

**DOI:** 10.1186/s12974-021-02175-2

**Published:** 2021-06-14

**Authors:** Li-Ming Chen, Chun-Hui Bao, Yu Wu, Shi-Hua Liang, Di Wang, Lu-Yi Wu, Yan Huang, Hui-Rong Liu, Huan-Gan Wu

**Affiliations:** 1 Yueyang Hospital of Integrated Chinese and Western Medicine, Shanghai University of Traditional Chinese Medicine, No.110 Ganhe Road, Shanghai, 200437 China; 2 Key Laboratory of Acupuncture and Immunological Effects, Shanghai University of Traditional Chinese Medicine, No. 650 South Wanping Road, Shanghai, 200030 China; 3 Faculty of Economics and Business, University of Groningen, Nettelbosje 2, Groningen, 9747 AE The Netherlands

**Keywords:** Inflammatory bowel disease, Depression, Tryptophan-kynurenine metabolic pathway, IDO, The brain-gut axis

## Abstract

Inflammatory bowel disease (IBD), which mainly includes ulcerative colitis (UC) and Crohn's disease (CD), is a group of chronic bowel diseases that are characterized by abdominal pain, diarrhea, and bloody stools. IBD is strongly associated with depression, and its patients have a higher incidence of depression than the general population. Depression also adversely affects the quality of life and disease prognosis of patients with IBD. The tryptophan-kynurenine metabolic pathway degrades more than 90% of tryptophan (TRP) throughout the body, with indoleamine 2,3-dioxygenase (IDO), the key metabolic enzyme, being activated in the inflammatory environment. A series of metabolites of the pathway are neurologically active, among which kynerunic acid (KYNA) and quinolinic acid (QUIN) are molecules of great interest in recent studies on the mechanisms of inflammation-induced depression. In this review, the relationship between depression in IBD and the tryptophan-kynurenine metabolic pathway is overviewed in the light of recent publications.

## Introduction

Inflammatory bowel disease (IBD) is a group of chronic inflammatory autoimmune diseases that primarily affect the gastrointestinal tract. The main symptoms of IBD include abdominal pain, diarrhea, bloody stools, and mucus stools. In severe cases, malnutrition and intestinal perforation may occur. IBD primarily includes two different classifications of ulcerative colitis (UC) and Crohn’s disease (CD), which affect approximately 5 million people worldwide. There is currently no curative treatment available for IBD [1]. At present, the prevalence of IBD in China is gradually increasing [2–4]. This increased prevalence may be linked to the changing environment, the Westernization of diet and lifestyle, and the resulting shift in the gut microbiota [5]. The etiology of IBD is a complex interplay of genetic, environmental, dietary, infectious, psychological, and other factors [6], and its pathogenetic repercussions are thought to be related to disordered gut-brain dialog, with psychosocial factors, as well as the gut microbiota that are involved in the process. In recent years, a growing body of clinical evidence has been published, demonstrating that IBD is strongly associated with depression. Patients with IBD have a higher prevalence of depression than the general population, and this higher prevalence is particularly pronounced in patients with active disease [7, 8]. Bidirectional gut-brain interactions have been widely accepted to explain the mechanism underlying the interactive effects of IBD and depression [9].

Tryptophan (TRP) metabolism plays an important role in the mechanisms associated with the gut-brain axis [10]. At least 90% of human intake of TRP is converted to kynurenine for further metabolism, a branch process known as the kynurenine pathway (KP). The remainder of TRP is metabolized to serotonin and indole [11]. Clinical studies have shown that TRP metabolism is associated with the severity of IBD [12]. In parallel, dysregulation of TRP metabolites such as serotonin, quinolinic acid (QUIN), and kynerunic acid (KYNA) has been linked to depressive behavior in animal models as well as human. Serotonin is associated with the gut microbiota and the gut-brain axis [10], while more importantly the IBD immune response has a significant impact on KP metabolism. The immune system is involved in the regulation of KP by influencing the activities of several enzymes, and downstream of these reactions, a variety of active substances are considered to be relevant to neural activity. In this review, the relationship between the key enzymes, products in KP, and depression in IBD will be summarized with reference to recent publications.

## The tryptophan-kynurenine pathway

**Fig. 1:**
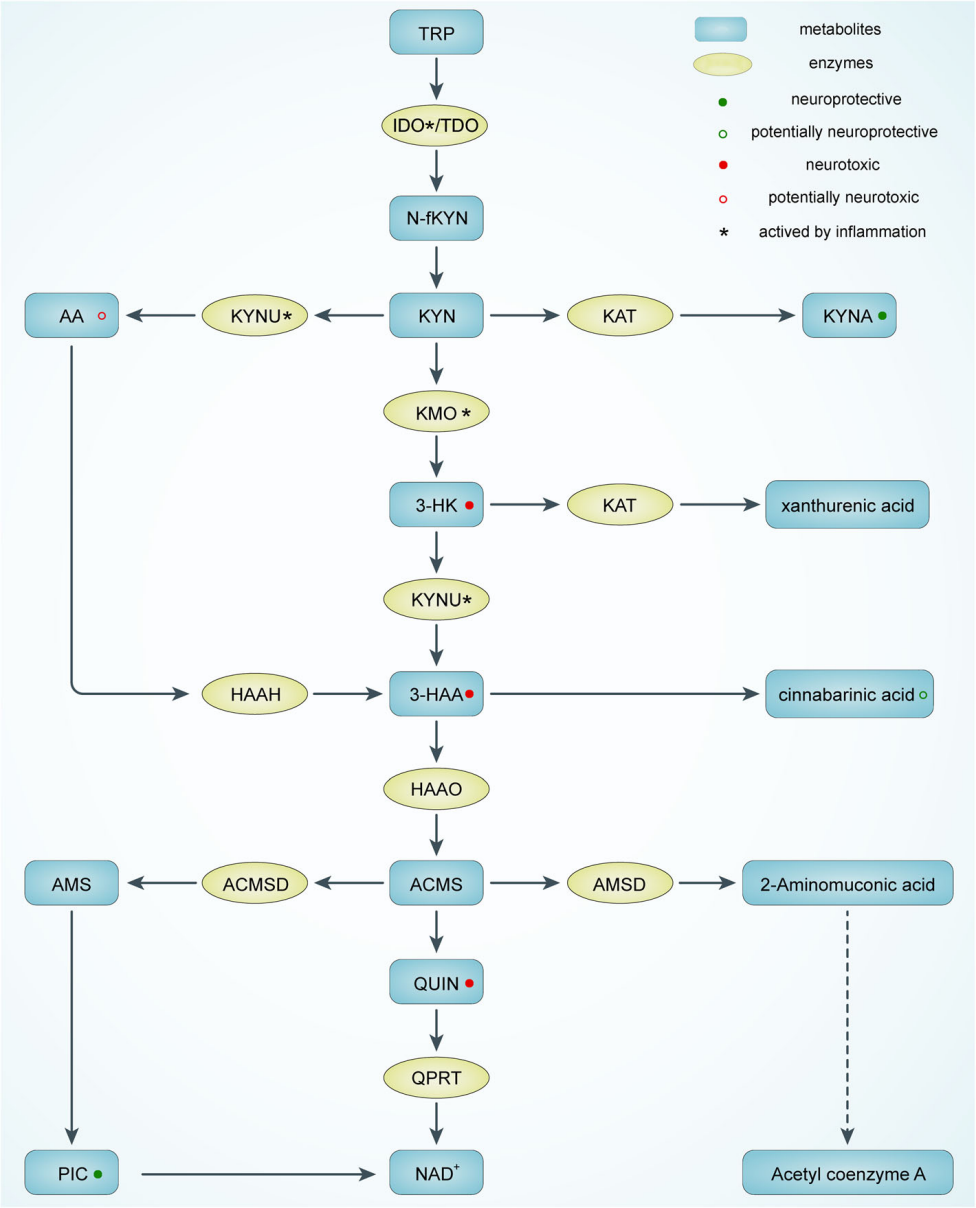
The tryptophan-kynurenine metabolic pathway. TRP, tryptophan; IDO, indoleamine 2,3-dioxygenase; TDO, tryptophan 2,3-dioxygenase; N-fKYN, N-formyl-kynurenine; AA, anthranilate acid; KYNU, kynureninase; KYN, kynurenine; KAT, kynurenine aminotransferase; KYNA, kynerunic acid; KMO, kynurenine 3-monooxygenase; 3-HK, 3-hydroxykynurenine; HAAH, 3-hydroxyanthranilic acid 3,4-hydroxylase; 3-HAA, 3-hydroxyanthranilic acid; HAAO, 3-hydroxyanthranilicacid 3,4-dioxygenase; AMS, 2-aminomuconic-6-semialdehyde; ACMSD, 2-amino-3-carboxymuconate-6-semialdehydedecarboxylase; ACMS, 2-amino-3-carboxymuconate-6-semialdehyde; AMSD, 2-aminomuconic-6-semialdehyde dehydrogenase; QUIN, quinolinic acid; QPRT, quinolinate phosphoribosyltransferase; PIC, picolinic acid; NAD^+^, nicotinamide-adenine-dinucleotide

TRP is one of the eight essential amino acids in the human body and is metabolized mainly in the liver. In organs, the presence of TRP ranges from the brain, kidney, and skeletal muscle [11]. The intestinal environment includes intestinal epithelial cells, chromophores, and microbiota, mediating the transformation and biochemical degradation of some intestinal TRPs [13]. Dietary TRP enters the body and is degraded through three main pathways: the serotonin pathway via tryptophan hydroxylase 1 conversion, the tryptophan-indole pathway (which activates aromatic hydrocarbon receptors and has four subpathways), and the KP pathway via the conversion of indoleamine 2,3-dioxygenase (IDO) and tryptophan-2,3-dioxygenase (TDO) (Fig. 1) [13]. The vast majority of human TRP intake is transformed through KP.

KP is initiated by the conversion of TRP to N-formylkynurenine (N-fKYN) in the presence of two rate-limiting enzymes, IDO, and TDO. N-fKYN is an unstable product that is immediately converted to kynurenine (KYN) by kynurenine formamidase. TDO has now been demonstrated to primarily mediate the basal metabolism of KP, whereas IDO functions more in an immune-activated environment [14]. KYN, the first stable product of KP, is the central node of the pathway. Three major transformation pathways diverge from KYN: (A) being deaminated to KYNA through kynurenine aminotransferase (KAT), (B) being degraded to anthranilic acid (AA) by kynureninase (KYNU), and (C) being converted to 3-hydroxykynurenine (3-HK) via kynurenine 3-monooxygenase (KMO).

Then, 3-HK is partially converted to 3-hydroxyanthranilic acid (3-HAA) by KYNU and partially to xanthurenic acid (XA) by KAT. A part of AA is also changed to 3-HAA. In rat brain tissue, AA is a more efficient precursor of 3-HAA [15]. A portion of the 3-HAA then generates cinnabarinic acid (CA), and the other portion then goes through 3-hydroxyanthranilic acid 3,4-dioxygenase (HAAO) to generate 2-amino-3-carboxymuconate-6-semialdehyde (ACMS), a precursor that is metabolized to QUIN under physiological conditions, which further generates nicotinamide-adenine-dinucleotide (NAD^+^) via quinolinate phosphoribosyltransferase (QPRT). In the other two metabolic branches, ACMS can be generated as picolinic acid (PIC) via the intermediate 2-aminomuconic-6-semialdehyde (AMS) in the presence of 2-amino3-carboxymuconate-6-semialdehydedecarboxylase (ACMSD), as 2-aminoglycoside acid catalyzed by 2-aminomuconic-6-semialdehyde dehydrogenase (AMSD) and finally as acetyl coenzyme A through a series of biochemical reactions. All metabolites are collectively referred to as “kynurenergic” substances.

## IBD and depression

Strong emphasis has been placed on the relationship between gastrointestinal diseases and emotional factors with the development of the “biopsychosocial” model of medicine. The inflammatory bowel disease questionnaire (IBDQ) [16], developed in 1989, includes emotional factors as one of its four dimensions. Depression and anxiety are global health problems and often accompany illness [17]. Stress is a common cause of early depression [18]. These three associated factors are usually accompanied by each other in IBD.

Since the turn of the twenty-first century, more clinical studies have demonstrated a strong link of IBD to both depressed mood and even the onset of depression. First, patients with IBD are more likely to develop mood disorders such as depression. Clinical studies in different countries have confirmed that the prevalence of anxiety and depression is higher in IBD patients than in the general population. For instance, in Canada, the combined results of two national surveys reported a prevalence of depression of 16.3% in the Canadian IBD population, of which 30% had considered suicide [19]. The Canadian IBD cohort study suggested that patients had higher lifetime prevalence rates of generalized anxiety disorder (GAD) and major depressive disorder (MDD) than the general population at 13.4% and 27.2% [20]. This is consistent with similar results which were found in studies initiated in the USA [7], China [21], Italy [22], Ireland [23], and South Korea [24]. A systematic review that combined multiple clinical studies showed that IBD had 19.2% and 21.2% anxiety and 21.2% depression, almost twice the rate of the general population, and 66.4% anxious mood and 34.7% depression in IBD patients who were also active. CD appeared to have a higher risk of emotional disorders than UC [25], and another systematic review also found similar results [26]. Most studies have concluded that women with IBD are more susceptible to anxiety and depression, while some studies do not support this conclusion [8]. The influence of gender on depression in IBD still needs further discussion. IBD-induced depression and other psychiatric problems affect the development of children and adolescents [27]. These problems need to be prevented since they may induce the risk of suicide in the elderly [28].

Furthermore, depression itself influences the occurrence and development of IBD. A review of studies based on UK electronic medical records investigating 403,665 cases of depression with 532,986 individuals with no history of depression reported a significantly increased risk of CD and UC in depressed patients [29]. Anxiety and depression increase the recurrence rate of IBD [30, 31]. Depression not only increases the risk of surgery or hospitalization in IBD patients [32] but also reduces postoperative ostomy recovery [33] and affects the effectiveness of anti-TNF therapy in CD patients [34]. In addition, anxiety is a better predictor of poor outcome in IBD than depression [35]. Depression and anxiety also alleviate the risk of fatigue in IBD patients, which in turn reduces their quality of life [36]. For IBD patients already in remission, depression is a factor in the development of irritable bowel-like symptoms [37]. Stressful life events are among the risk factors for IBD attacks [38], and maintaining a balanced and regular lifestyle may alleviate the severity of IBD [39]. Good sleep and exercise can help alleviate depression and reduce IBD episodes, while unhealthy lifestyle habits, such as smoking and alcohol consumption, invite a poor outcome of IBD [39]. Furthermore, nonadherence to treatment in IBD patients is strongly associated with disease relapse. Psychological factors, especially anxiety and depression, are among the focal points of research on treatment adherence in IBD patients [40], and most studies have concluded that there is a correlation between the two [41–44]. In studies of mesalazine [45], azathioprine, and anti-TNF [46, 47] for IBD, depression was found to be an important factor in nonadherence to medication, whereas a study of 356 IBD patients did not find an association between anxiety, depression, and treatment adherence, suggesting that further exploration is needed [48].

Finally, antidepressant treatment, including medication and psychotherapy, may have an ameliorative effect on depression in IBD [49]. Antidepressant medications are currently taken by 10–30% of IBD patients in Western countries, but the available evidence is still insufficient to support the improvement in the condition and quality of life of IBD patients with these medications [50, 51]. Based on epidemiological investigations, drugs such as selective serotonin reuptake inhibitors and tricyclic antidepressants have been shown to be protective against IBD [29]. Current clinical controlled trials support that tianeptine (for UC) [52] and duloxetine (for IBD) [53] may simultaneously reduce anxiety and depression scores as well as the disease activity index in patients, while fluoxetine [54] showed no benefit, but the low sample size may limit the reliability of these studies. A meta-analysis [55] evaluated 14 psychological treatments for IBD and found that patients with IBD in remission showed significant improvements in depression scores and quality of life at the end of treatment, but this improvement was not sustained over time, and the effect was not significant for active IBD. Treatment of IBD inflammation also alters the depressive state of the patient. Anti-TNF therapy and immunosuppressants can significantly reduce depression in IBD patients [56], and anti-TNF therapy may reduce anxiety and depressive symptoms by modulating central nervous function and improving cognitive-emotional processing in IBD patients [57]. Vedolizumab improves both sleep and depression in IBD patients [58].

The etiology of depression in IBD includes both external and somatic factors. A cross-sectional investigation indicated that lack of social support and increasing disease activity are both independent correlators with IBD depression [59]. At the level of external factors, the condition of IBD has been suggested to enhance insecurity of patients and alter their psychological attachment style, thus creating chronic stress and leading to an impact on their mental health [60]. At the somatic level, the pathological effects of IBD may disrupt the neurological function of the patients by themselves. With deepening research on gut-brain interactions, diseases with obvious features of gut-brain dysfunction, such as irritable bowel syndrome and IBD, have recently been proposed to be called disorders of “gut-brain interaction (DGBI)” [61]. The pathology of these diseases generally consists of both central nervous disorders and gut dysfunction, the latter further including peristaltic disorder, allergic reactions, mucosal inflammation, alterations in immune and microbial integrity, etc. Gracie et al. [62] presented a clinical trial validating the relationship between IBD activity and the bidirectional gut-brain and brain-gut exacerbation of anxiety and depression. These studies have expanded the knowledge of depression in IBD, and thus, depression has been suggested to be an extraintestinal manifestation of IBD inflammation [63]. Many current assessments of anxiety and depression in IBD have been suggested to be methodologically problematic [64, 65]. The Hospital Anxiety and Depression Scale (HADS), which has been widely used in IBD depression-related studies, has some limitations in sensitivity, whereas the Patient Health Questionnaire (PHQ-9) may ameliorate these problems [64, 66]. Moreover, there is little evidence that corticosteroids induce depression in patients with IBD [67, 68]. Therefore, future relevant clinical studies need to place more emphasis on medication and other demographics of the patients [65].

## KP and gut-brain axis metabolism in IBD

### KP and intestinal inflammation in IBD

A multilevel association exists between KP and IBD. Administration of TRP and some of its metabolites to a mouse model of colitis has been found to reduce the severity of colitis. Removal of TRP from the diet increases the susceptibility of mice to colitis [69, 70]. The gut microbiota phenotype of TRP-deficient mice can be transferred to normally nourished mice by transplantation of microbiota [71]. Several animal studies have confirmed that TRP promotes the remission of experimental colitis by modulating intestinal protein turnover [72], mucosal permeability [73], immune response [74], and gut microbiota [75]. Studies on IBD patients have also confirmed that their serum TRP levels were also significantly lower than the serum TRP levels of normal controls, and serum TRP levels in IBD patients were negatively correlated with disease activity and C-reactive protein levels, along with a greater decrease in CD than UC, which may be related to aberrant intake and metabolism of TRP in IBD patients. Low TRP levels may also serve as a predictor of IBD surgery [12].

Since diet is a factor in the progression of IBD, IBD patients are often advised by physicians to change their dietary habits [76]. From the end of TRP absorption, it is unclear what effect dietary changes in IBD patients will have on TRP intake. Although serum TRP levels in CD patients are lower than serum TRP levels in UC patients, no difference in dietary TRP intake between the two diseases has been found in recipe surveys [12]. TRP is ingested via the sodium-dependent neutral amino acid transporter protein family 6 member SLC6A19/B^0^AT1 [71], which is predominantly expressed in the small intestine in humans and rodents [77]. B^0^AT1 messenger RNA was found to be significantly lower in colon biopsies from IBD patients than in controls, suggesting that IBD patients may have a specific TRP uptake disorder [12].

At the metabolic end of TRP, more than 90% of TRP is degraded by IDO/TDO-mediated KP, while the remainder is metabolized to serotonin or generated as indole derivatives via the gut microbiota [13]. The findings in colon biopsies of IBD patients with IDO messenger RNA and KYN/TRP [78] were considerably elevated, indicating that IDO is activated and accelerates the metabolism of TRP to KYN, further reducing the serum TRP reserve [12], which may be due to the activation of IDO by increased proinflammatory cytokines in IBD, including IFN-γ, IL-1, and IL-6 [79]. Several studies have supported that KYN/TRP is associated with important biomarkers of disease activity, C-reactive protein, sedimentation rate [80], and endoscopic score [81]; thus, KYN/TRP may be used as one of the biomarkers for the evaluation of IBD activity.

The aryl hydrocarbon receptor (AhR) is currently considered a potential target for the control of intestinal inflammation [75], with multiple metabolites of TRP (dominated by the gut microbiota-indole pathway) being its ligands [82]. In the presence of overactivation of IDO, reduced gastrointestinal TRP availability may result in a decrease in AhR ligands, which are produced by the gut microbiota. A similar situation occurs in the setting of intestinal inflammation such as DSS-induced colitis in mice, where supplementation with dietary TRP can reduce the severity of colitis by restoring AhR ligand production in the gut microbiota compartment [83]. A comparable protective effect of TRP was observed in a porcine model of colitis [84].

IDO may also have a physiological effect in controlling intestinal inflammation. Takamatsu et al. [85] knocked out IDO1 in mice, resulting in a more severe inflammatory response to TNBS-treated colitis. The pathological damage caused by IDO1 deficiency is partly due to the activation of proinflammatory cytokines and a decrease in the number of CD4^+^Foxp3^+^ regulatory T cells in the colon. However, Shon et al. [86] found that DSS-induced intestinal inflammation in IDO1 knockout mice was less severe than DSS-induced intestinal inflammation in wild-type mice. The discrepancy in conclusions may be due to the different mechanisms of modeling, and the specific mechanisms remain for further experimentation. Lee et al. [87] found that nonsynonymous single nucleotide polymorphisms of IDO1 in a small number of patients were associated with the occurrence of CD exacerbation, perianal disease, extraintestinal manifestations, and reduced serum KYN/TRP during the active phase, suggesting an association between hypofunction of IDO1 and increased inflammation in some patients with CD.

The gut microbiota play an important role in KP metabolism. Serum levels of tryptophan are lower in conventionally fed mice than in germ-free mice, indicating that the gut microbiota may metabolize a portion of tryptophan [88]. In addition to inducing the conversion of tryptophan to indole, evidence suggests that the gut microbiota have a role in regulating KP [10, 13], as demonstrated by the lower expression of IDO in the intestinal epithelium of germ-free mice [89]. As different bacteria carry different KP metabolism enzymes [90], we can hypothesize that KP is shared by multiple bacteria in the gut microbiota. *Escherichia coli* converts KYN to KYNA [91]. The concentration of KYNA in the intestinal lumen is much higher than the high concentration in the intestinal wall, which may be a combination of the gut microbiota and digestive fluid [91, 92]. KYNA is suggested to exhibit some bactericidal effects, and low-medium concentrations of KYNA in the digestive fluid also promote the growth of some probiotics [93]. Interestingly, KYNA in the intestinal lumen is thought to have two sides: promoting or inhibiting inflammation at the same time. On the one hand, KYNA activates G protein-coupled receptor 35 (GPR35) in the intestinal wall, which may exacerbate chronic stress and DSS-induced intestinal inflammation by affecting nod-like receptor protein 3 (NLRP3) [94]. On the other hand, KYNA is a broad-spectrum competitive antagonist of glutamate (Glu) receptors. KYNA antagonizes intestinal N-methyl-D-aspartate (NMDA) receptors, a type of Glu receptor. KYNA regulates oxidative and nitrosative stress pathways and reduces the production of inflammatory factors [95]. A recent meta-analysis showed that probiotics significantly affect serum KYN and KYN/TRP ratios, suggesting that probiotics can regulate KP [96]. A methyldeficiency diet upregulates KP, and probiotic supplementation relatively downregulates KP, especially reducing AA and 3-HK. Similar effects have been observed for multiple probiotic strains [97].

The subsequent metabolism of KYN was similarly altered during the pathology of IBD. KMO knockdown reduced intestinal inflammation in TNBS-infused rats, suggesting that KYN may have a protective effect on intestinal inflammation [98]. Among the patient population, significant decreases in PIC levels were observed in CD patients, and significant increases in serum QUIN levels were observed in both CD and UC patients [12]. KYN levels and serum KYNU expression were specifically decreased and 3-HAA specifically increased in ileal CD patients [99]. The results of serum KYNA levels have varied in different studies. Nikolaus et al. [12] found an overall decrease in KYNA levels in CD patients, while some other studies [100–102] found an increase in KYNA during the active phase of both types of IBD. The difference in results may be due to the variation in sample size. Overall, the KP metabolism of IBD patients is shifted towards QUIN compared to the healthy population.

### KP and inflammation-induced depression

The association between KP and depression is extensive and profound. Serotonin, one of the most essential metabolites of TRP, is involved in the central nervous system (CNS) in the regulation of mood, behavior, and cognitive function. As early as 1969, a review published in The Lancet proposed the “serotonin hypothesis” of depression, stating that increased blood levels of steroids lead to activation of hepatic TRP-pyrrolase (TDO), resulting in increased metabolism of the TRP-KP pathway and decreased production of serotonin, which affects neurological function. As the inhibitory effect of serotonin on the amygdala of the brain is diminished, this diminution leads to increased steroid production and a negative cycle [103]. Subsequently, selective serotonin reuptake inhibitors have been widely adopted as antidepressants, and this hypothesis has become a popular research direction in the field of depression [104].

After absorption of TRP through the intestinal tract, some of the TRP is metabolized to subsequent products through the intestine, liver, and other organs, and some of the TRP enters the circulation directly [9]. TRP, KYN, and other of their products can pass the blood-brain barrier [105]. In the intracerebral environment, IDO is expressed mainly on astrocytes and microglia. Approximately 40% of the KYN content is synthetically produced in the brain, and the rest is derived from plasma [106]. IDO has been proven to be activated by inflammatory cytokines such as IFN-γ and TNF-α. Researchers have found reduced circulating TRP levels in cancer patients treated with IL-2 or IFN-α [107], as well as elevated inflammatory cytokines in some depressed patients [108]. With this evidence, the concept of “inflammation-induced depression” was developed, and the “immune activation-mediated TRP depletion hypothesis” was proposed as a hypothesis for the etiology of inflammation-induced depression [109].

However, after a period of exploration, researchers found that direct TRP injections did not seem to improve inflammation-induced depressive symptoms. There was no direct evidence to support reduced TRP and serotonin levels in the brains of patients with inflammation-induced depression, and depressive symptoms appeared to lack a direct link to TRP availability [96]. Similar findings have been confirmed in clinical studies in CD [110]. A series of animal studies demonstrated that the breakdown products of TRP have powerful pharmacological effects on the brain [111], while IDO pharmacological inhibition or knockdown attenuated depressive behavior in rats [112, 113]. Researchers are beginning to focus their studies on inflammation-induced depression on the metabolites of KYN. When KYN is metabolized downstream, the production of KYNA occurs primarily in astrocytes, whereas 3-HK and its downstream product QUIN are produced primarily in microglia [114], and QUIN needs to travel to QPRT-containing astrocytes or neurons to be further metabolized into NAD^+^ [115].

The main metabolite of KP, QUIN, is an agonist of NMDA receptors. QUIN is a neuroexcitatory toxin that is found mainly in the forebrain [111]. Glu is the most abundant excitatory neurotransmitter in the brain and is essential for information processing, memory, and neuronal plasticity. Glu is interconverted with glutamine inside and outside the synapse through a series of mechanisms to form the glutamatergic system. NMDA receptors are one of the types of Glu receptors. Current research suggests that the series of neurotoxic effects produced by overactivation of NMDA receptors is one of the important mechanisms of depression [116]. QUIN lacks the capacity to cross the blood-brain barrier and requires local generation in the brain [105]. During KP metabolism in the CNS, the rate of QUIN generation by 3-HAO is much higher than the rate at which QPRT metabolizes QUIN, allowing QUIN to accumulate readily in brain regions and produce persistent stimulation of NMDA receptors [111], thereby damaging the corresponding neuronal cells and inducing depression. The hippocampus is a major component of the limbic system that plays a role in the regulation of mood, behavior, immunity, learning and memory, and cognitive functions. Damage to the hippocampus may be a mechanism for depression [117]. The damage affects both neurons and glial cells. NMDA receptor 2A and 2B subtypes have the highest affinity for QUIN and are widely distributed in the hippocampus. QPRT activity is low in the hippocampus, and QUIN is difficult to metabolize in the hippocampus; therefore, neurotoxic QUIN damages the hippocampus more easily [118]. Imaging studies also suggest that the increase in gray matter volume in the hippocampus and other emotion-generating brain regions of CD patients is closely related to changes in depression and anxiety [119], which may be related to immune activation prompting changes in glial cells [120]. This process presents a hypothesis for the depression-promoting mechanism of increased QUIN in the brain. In addition, the relevant targets of QUIN neurotoxicity involve energy dysfunction, oxidative stress, transcription factors, cytoskeletal disruption, behavioral changes, and cell death. These numerous factors may all be potential mechanisms by which QUIN promotes depression.

KYNA is a broad-spectrum competitive antagonist of Glu receptors and an inhibitor of α7 nicotinic acetylcholine receptors (α7nAChRs). Due to its polarity, KYNA in the circulation rarely crosses the blood-brain barrier into the CNS, and KYNA in the CNS is generated mainly by local metabolism of KYN [121]. α7nAChRs are major targets of KYNA in the brain, mediating the bidirectional effects of KYNA on neurotransmitter levels. In different regions of the forebrain, a modest increase in KYNA leads to a rapid decrease in extracellular Glu and dopamine levels, whereas inhibition of KYNA synthesis increases these neurotransmitter and acetylcholine levels [111]. As a consequence, KYNA exhibits certain neuroprotective effects and may have the potential to alleviate depression.

In terms of clinical evidence, a recent meta-analysis of 22 studies summarizing the clinical situation of a total of 1894 patients with depression and their KP metabolites of KYN, KYNA, and QUIN found that depressed patients had reduced levels of KYNA and KYN and increased levels of QUIN in patients not on antidepressants [122]. The vast majority of the studies tested the serum or plasma of the patients. Three groups of studies directly analyzed the levels of relevant metabolites in human brain tissue and cerebrospinal fluid: Clark et al. [123] detected ventral lateral frontal cortex tissue from 45 patients who passed away from various causes of depression and found reduced expression of TDO and IDO messenger RNA and lower levels of QUIN compared to controls. A cerebrospinal fluid study of 64 suicide attempts by Erhardt et al. [124] found that QUIN was elevated and KYNA was decreased. The increase in QUIN was associated with cerebrospinal fluid IL-6 levels. Another group of autopsy studies found that QUIN levels were higher in the anterior cingulate cortex and midbrain cortical microglia in patients who died by suicide from depression [125]. The different findings may be related to differences in study methodology. Studies on the association of KP metabolites in the peripheral blood and CNS are scarce, and only one study that included 16 patients receiving IFN-α for hepatitis C showed that peripheral and QUIN levels in the CNS were highly correlated after IFN-α-induced IDO activation [126].

Several studies identified KYNA/QUIN as a putative neuroprotective factor that was lower in depressed patients and negatively correlated with low pleasure and positively correlated with the volume of the hippocampus and amygdala in depressed patients [127, 128]. Ketamine, as an NMDA receptor antagonist, has shown high efficacy in depression. While antagonizing the action of QUIN, ketamine can act on brain microglia to reduce QUIN production. Baseline KYNA/QUIN may serve as a predictor of the response to ketamine in drug-resistant depression [120]. However, ketamine is also an addictive drug, for which further promotion is limited.

The gut microbiota have an impact on KP in the CNS and are involved in the process of psychiatric disorders such as depression [129]. Experimental animals subjected to different stresses have increased brain and intestinal KYN/TRP ratios and upregulated IDO expression, accompanied by changes in the gut microbiota [130, 131]. Transplantation of gut microbiota from depressed patients to experimental animals increases depressive behavior, the KYN/TRP ratio and serum C-reactive protein in the animals [132, 133]. Small sample clinical studies suggest that fecal bacteria transplantation may alleviate depressive symptoms in IBD [134]. Probiotics can be used to assist in the treatment of depression [135]. As mentioned above, both preclinical and clinical studies revealed that a variety of probiotics lower the KYN/TRP ratio while alleviating depressive symptoms [136–138].

The field of metabolic enzymes of KP, in addition to IDO/TDO, decreased activity of KAT [139], KMO activation [140], and single nucleotide polymorphisms of KMO and KATIII [141], are also associated with depression; however, the scale of the study is still limited. ACMSD is a key enzyme in KP for the generation of QUIN and mediates the synthesis of another product, PIC. As one of the products of KP can be excreted directly from the body, the role of PIC is currently poorly understood [142]. A clinical study of suicide attempts shows a decrease in cerebrospinal fluid and blood PIC and a simultaneous decrease in the PIC/QUIN ratio, and the SNP rs2121337 minor C allele of ACMSD, associated with an increase in cerebrospinal fluid QUIN, is more prevalent in suicide attempts [143]. Other intermediate products of KP, such as 3-HK [144], 3-HAA [145] and AA, have been found to be involved in complex pro-oxidant and antioxidant processes in the CNS [111], which may cause neuronal damage. Some byproducts of the KP such as XA and CA have been found to be neuroactive [146]. Research on the specific relationship between these products and inflammation-related depression remains to be expanded.

## Conclusions

**Fig. 2:**
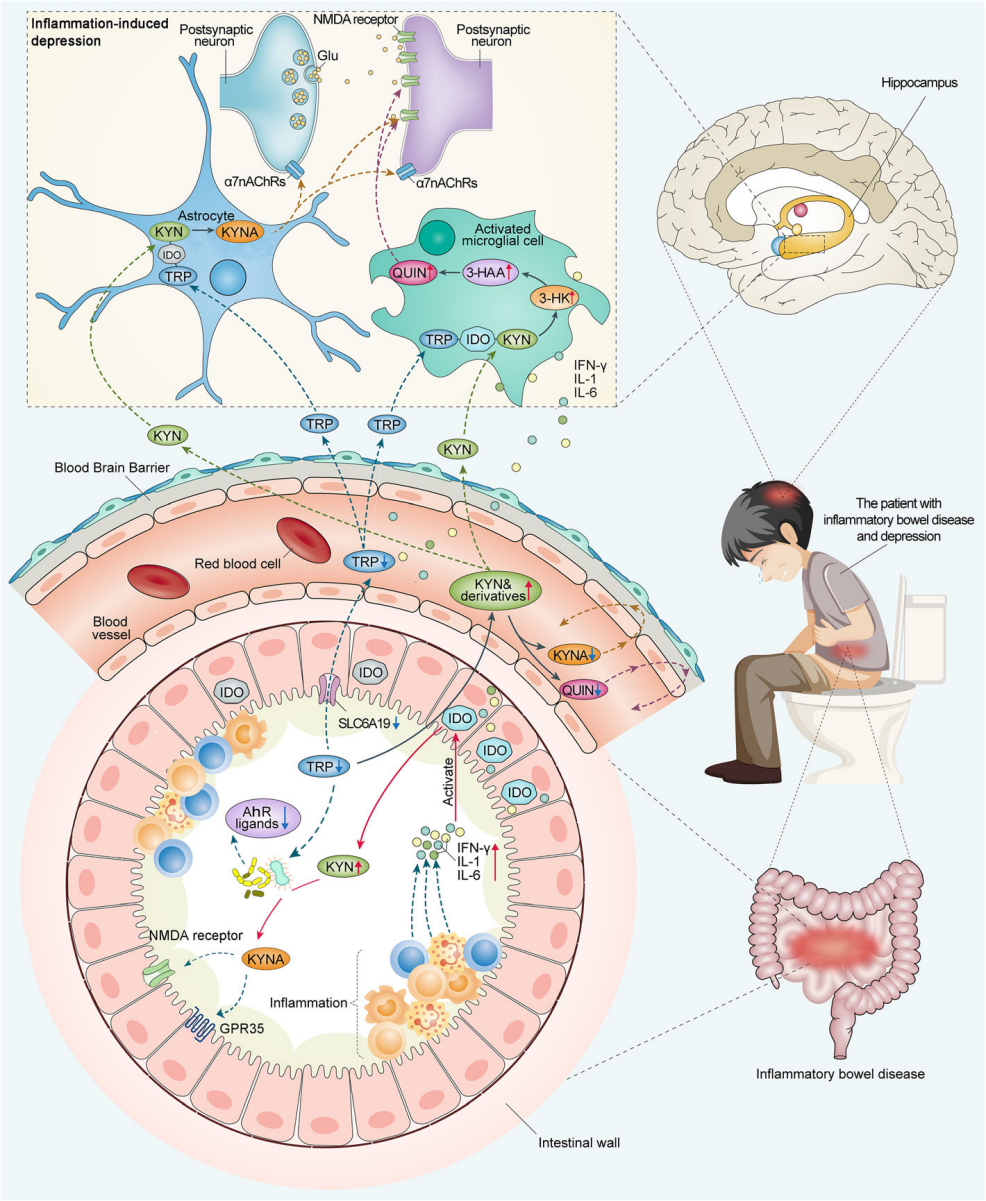
The link between intestinal inflammation, KP, and depression in IBD. In the process of IBD, inflammatory activity stimulates intestinal cells to produce a series of inflammatory cytokines such as IFN-γ, IL-6, and IL-1. Activation of IDO by inflammatory cytokines results in increased degradation of TRP to KYN, which crosses the blood-brain barrier and is metabolized by different branches to QUIN and KYNA. In an inflammatory environment, a higher rate of production of neurotoxic molecules such as QUIN, 3-HK, and 3-HAA may cause depression by damaging hippocampal neurons. In contrast, KYNA is a neuroprotective factor. These KP metabolites affect the mood of IBD patients through a complex series of neurobiological responses. IFN, interferon; IL, interleukin; α7nAChRs, α7 nicotinic acetylcholine receptors; Glu, glutamate; NMDA, N-methyl-D-aspartate

As one of the mechanisms of depression, KP metabolism disorder connects two important etiology hypotheses of depression: “cytokine hypothesis” and “receptor hypothesis”. Due to its impact on the hippocampus, the two important etiology hypotheses of depression are also related to the “neuroplasticity hypothesis” [147]. As mentioned above, depression in IBD may arise through an “IBD-inflammation-KP-depression” association (Fig. 2). The core mechanism may be the overactivation of IDO throughout the body and in the brain due to chronic inflammation, which increases the metabolic rate of KP and ultimately generates excess QUIN and less KYNA in the brain. The neurotoxicity of QUIN affects glial cells and neurons, resulting in inflammation-induced depression. KYNA has a neuroprotective effect, and KYNA/QUIN can be considered a neuroprotective index. Regarded as a “bottom-up” pathway in the gut-brain interaction, this metabolic pathway begins in the gut and ends in the hippocampus and other brain structures. Preliminary evidence for this pathway has been found in experimental animals [148] and in patients with IBD [12]. Regarding the issues of IBD inflammation affecting KP in the CNS, IDO activation is a strong focus in the current literature, while unknown aspects such as the activation and expression of other key KP enzymes such as KMO, KAT, and ACMSD remain to be explored. The involvement of KP intermediates in CNS oxidative and antioxidative stress needs to be revealed in more detail. These downstream metabolic factors may be more important than IDO in the regulation of KP and should attract more attention from researchers in the next phase of research, which will help in more precise management of inflammation and psychological conditions in IBD patients.

## Data Availability

Not applicable.

## Abbreviations

3-HAA: 3-Hydroxyanthranilic acid
3-HK: 3-Hydroxykynurenine
α7nAChRs: α7 Nicotinic acetylcholine receptors
AA: Anthranilate acid
ACMS: 2-Amino-3-carboxymuconate-6-semialdehyde
ACMSD: 2-Amino-3-carboxymuconate-6-semialdehydedecarboxylase
AhR: Aryl Hydrocarbon Receptor
AMS: 2-Aminomuconic-6-semialdehyde
AMSD: 2-Aminomuconic-6-semialdehyde dehydrogenase
CA: Cinnabarinic acid
CD: Crohn’s disease
CNS: Central nervous system
DGBI: Disorders of gut-brain interaction
DSS: Dextran sodium sulfate
GAD: Generalized anxiety disorder
Glu: Glutamate
GPR35: G protein-coupled receptor 35
HAAO: 3-Hydroxyanthranilicacid 3,4-dioxygenase
HADS: The Hospital Anxiety and Depression Scale
IBD: Inflammatory bowel disease
IBDQ: The inflammatory bowel disease questionnaire
IDO: Indoleamine 2,3-dioxygenase
IFN: Interferon
IL: Interleukin
KAT: Kynurenine aminotransferase
KMO: Kynurenine 3-Monooxygenase
KP: Kynurenine pathway
KYN: Kynurenine
KYNA: Kynerunic acid
KYNU: Kynureninase
MDD: Major depressive disorder
N-fKYN: N-formyl-kynurenine
NAD^+^: Nicotinamide-adenine-dinucleotide
NMDA: N-methyl-D-aspartate
NLRP3: Nod-like receptor protein 3
PHQ-9: The Patient Health Questionnaire 9
PIC: Picolinic acid
QPRT: Quinolinate phosphoribosyltransferase
QUIN: Quinolinic acid
SLC6A19/B^0^AT1: Solute carrier family 6 member 19/system B(0) neutral amino acid transporter 1
TDO: Tryptophan 2,3-dioxygenase
TNBS: 2,4,6-Trinitrobenzenesulfonic acid
TNF: Tumor necrosis factor
TRP: Tryptophan
UC: Ulcerative colitis
XA: Xanthurenic acid
